# The Histone Deacetylase Inhibitor Romidepsin Spares Normal Tissues While Acting as an Effective Radiosensitizer in Bladder Tumors in Vivo

**DOI:** 10.1016/j.ijrobp.2020.01.015

**Published:** 2020-05-01

**Authors:** Salome Paillas, Chee K. Then, Susan Kilgas, Jia-Ling Ruan, James Thompson, Amy Elliott, Sean Smart, Anne E. Kiltie

**Affiliations:** Department of Oncology, CRUK/MRC Oxford Institute for Radiation Oncology, University of Oxford, United Kingdom

## Abstract

**Purpose:**

Muscle-invasive bladder cancer has a 40% to 60% 5-year survival rate with radical treatment by surgical removal of the bladder or radiation therapy–based bladder preservation techniques, including concurrent chemoradiation. Elderly patients cannot tolerate current chemoradiation therapy regimens and often receive only radiation therapy, which is less effective. We urgently need effective chemotherapy agents for use with radiation therapy combinations that are nontoxic to normal tissues and tolerated by elderly patients.

**Methods and Materials:**

We have identified histone deacetylase (HDAC) inhibitors as promising agents to study. Pan-HDAC inhibition, using panobinostat, is a good strategy for radiosensitization, but more selective agents may be more useful radiosensitizers in a clinical setting, resulting in fewer systemic side effects. Herein, we study the HDAC class I-selective agent romidepsin, which we predict to have fewer off-target effects than panobinostat while maintaining an effective level of tumor radiosensitization.

**Results:**

In vitro effects of romidepsin were assessed by clonogenic assay and showed that romidepsin was effective in the nanomolar range in different bladder cancer cells and radiosensitized these cells. The radiosensitizing effect of romidepsin was confirmed in vivo using superficial xenografts. The drug/irradiation combination treatment resulted in significant tumor growth delay but did not increase the severity of acute (3.75 days) intestinal normal tissue toxicity or late toxicity at 29 weeks. Moreover, we showed that romidepsin treatment impaired both homologous recombination and nonhomologous end joining DNA repair pathways, suggesting that the disruption of DNA repair pathways caused by romidepsin is a key mechanism for its radiosensitizing effect in bladder cancer cells.

**Conclusions:**

This study demonstrates that romidepsin is an effective radiosensitizer in vitro and in vivo and does not increase the acute and late toxicity after ionizing radiation. Romidepsin is already in clinical use for the cutaneous T-cell lymphoma, but a phase 1 clinical trial of romidepsin as a radiosensitizer could be considered in muscle-invasive bladder cancer.

**Summary**

The class I histone deacetylase inhibitor romidepsin sensitizes bladder cancer cells to ionizing radiation (IR) and delays tumor growth after IR. Treatment with romidepsin + IR did not increase the normal tissue toxicity caused by radiation to the surrounding normal bowel incorporated in the radiation field acutely at 3.75 days after radiation or later at 29 weeks. Romidepsin treatment impaired both homologous recombination and nonhomologous end joining DNA repair pathways.

## Introduction

Bladder cancer is the ninth most frequent malignancy and the 13th most common cause of death, worldwide. Bladder cancer is a highly prevalent disease and is associated with substantial morbidity, mortality, and cost.^1^ Approximately 70% of bladder tumors are nonmuscle invasive bladder cancers, and the rest are muscle-invasive bladder cancers (MIBCs). MIBC presents an unfavorable patient prognosis with a 5-year survival rate of <50%. The treatments available for MIBC are radical cystectomy, often preceded by neoadjuvant chemotherapy,^2^ or radiation therapy–based bladder preservation techniques, including concurrent radiosensitizing chemotherapy.^3^ Elderly patients cannot tolerate current chemoradiation therapy regimens and receive radiation therapy only, which is less effective.^4^, ^5^, ^6^ Therefore, an urgent need exists to find new radiosensitizers that are less toxic to normal tissues for this elderly patient population.

The high expression levels of histone deacetylases (HDACs) observed particularly in high-grade urothelial bladder cancer clearly warrant subsequent studies on the potential use of HDAC inhibitors (HDACi) as a novel therapeutic approach.^7^, ^8^, ^9^ HDACi exhibit low toxicity to normal cells,^10^ and we found the pan-HDACi panobinostat to be promising as a radiosensitizer in vitro^10^ and in vivo.^11^ Although pan-HDAC inhibition is a promising strategy for radiosensitization, more selective agents may have superior efficacy with fewer adverse effects. The class I selective HDACi romidepsin has not yet been used in bladder cancer but is approved by the U.S. Food and Drug Administration for the treatment of cutaneous T-cell lymphoma.^12^ Class I HDACs are known to be associated with an overexpression in urothelial cancer compared with normal urothelium^7^; thus, we hypothesized that romidepsin alone could have fewer off-target effects than panobinostat while maintaining an effective level of radiosensitization.

In this study, we demonstrate that treatment with romidepsin results in radiosensitization of bladder cancer cells in vitro and in vivo without showing any exacerbation of acute or late toxicity in the intestines and the bladder. Moreover, romidepsin treatment impaired both homologous recombination (HR) and nonhomologous end joining (NHEJ) DNA repair pathways, suggesting a key mechanism for its effects of radiosensitization in bladder cancer cells.

## Methods and Materials

All animal work was done in accordance with United Kingdom Home Office Guidelines, per the Animal Research: Reporting of In Vivo Experiments guidelines, and approved by the University of Oxford Animal Welfare and Ethical Review Body under University of Oxford project licenses P4B738A3B and P8484EDAE. Group sizes were chosen to detect large effect sizes by using a G-Power analysis program. All mice were purchased from Charles Rivers UK Ltd.

### Cell lines, drugs, and irradiator

All cell lines were obtained from the American Type Culture Collection. RT112, MBT2, and HT1376 cells were grown in RPMI-1640 medium (Sigma), supplemented with 10% fetal bovine serum (Invitrogen). DR-GFP U2OS cells were kindly provided by Dr Sovan Sarkar of the University of Oxford; cells were grown in Dulbecco's modified eagle medium and supplemented with 10% fetal bovine serum. All cell lines were tested for mycoplasma and found to be negative.

Romidepsin was purchased from Stratech Scientific Ltd (S3020-SEL; Cambridge, United Kingdom) and used in 5% DMSO and dH_2_O. DMSO was purchased from Sigma-Aldrich (D2650; Gillingham, United Kingdom).

Cells were irradiated in complete medium at a dose rate of 1.5 Gy/min using the Gamma-Service Medical GmbH GSR D1 irradiator.

### Colony formation assay

Cells were seeded in 5 cm dishes at appropriate densities in triplicate, treated with DMSO or romidepsin at appropriate concentrations for 24 hours, and irradiated with 0, 2, 4, 6, and 8 Gy. Cells were cultured for 10 to 12 days, and colonies were counted as described in the supplementary data.

### Xenograft model for growth delay and survival studies

RT112 cells were prepared in phenol red-free Matrigel (BD Biosciences) and phosphate-buffered saline 1:1; 100 μL (5 × 10^6^ cells) was injected into the flank of 6- to 7-week-old female CD1-nude mice. Mice were randomized in Excel (Microsoft) using RAND function into 4 groups: vehicle (5% DMSO in dH_2_O), romidepsin (4 mg/kg, single dose, intraperitoneally), IR (using a Gulmay-320 cabinet, 6 Gy, single dose), and romidepsin + IR (6 hours after romidepsin treatment). They received the corresponding treatment when the tumors reached 50 mm^3^. Tumors were measured 3-dimensionally 3 times a week with a manual calipers, and tumor volume was calculated using the formula (width × length × height × [π/6]). Mice were sacrificed when the tumors reached the limit size of 350 mm^3^.

### Normal tissue response models

#### Acute toxicity

The acute toxicity was assessed as described in Methods E1 (available online at https://doi.org/10.1016/j.ijrobp.2020.01.015).

#### Long-term toxicity

The long-term toxicity was assessed as described in Methods E1 (available online at https://doi.org/10.1016/j.ijrobp.2020.01.015).

#### Crypt assay

The crypt assay was performed as described in Methods E1 (available online at https://doi.org/10.1016/j.ijrobp.2020.01.015).

#### Western blots

Western blot samples were prepared as previously described.^13^ Protein visualization was performed using the following antibodies: H3K18Ac (Cell Signaling Technology, #9675), Phospho-Histone H2A.X Ser139 (Millipore, #2739172), and βActin (Abcam, #A1978), and an infrared LiCor Odyssey imaging system (LiCor Biosciences). All Western blots were performed twice independently.

#### Homologous recombination assays

To measure HR efficiency, 8 × 10^5^ U2OS DR-GFP cells were seeded in 6-well plates. Cells were treated with siLuciferase or siRAD51 for 48 hours and then transfected with 4 μg of I-SceI plasmid using Lipofectamine 3000 per the manufacturer’s instructions (Invitrogen) for 24 hours. Subsequently, romidepsin or vehicle was added for 24 hours. Cells were recovered in a fresh complete medium for at least 24 hours and GFP-positive cells were determined using BD FACS DIVA software. The data were then normalized to the I-SceI positive control and analyzed using FlowJo V10. Statistical significance was determined using a one-way analysis of variance (ANOVA). All experiments were conducted in duplicate across 3 technical replicates.

#### Nonhomologous end joining assays

U2OS NHEJ reporter cells were seeded in 6-well plates. The following day, cells were treated with DMSO, romidepsin (25 nM for 24 hours), or DNPA-PK inhibitor NU77441 (2 μM for 2 hours) and transfected with 5 μg of I-SceI plasmid (for at least 8 hours) using Lipofectamine 3000 per the manufacturer’s instructions (Invitrogen). Cells were recovered after treatment in complete medium for at least 24 hours, and GFP-positive cells were quantified using BD FACS DIVA software. The data were normalized to I-SceI positive control and analyzed using FlowJo V10. All experiments were conducted in triplicate across 2 technical replicates.

### Statistical analysis

All statistical analyses were performed using GraphPad Prism 8 software unless specified otherwise. All data are representative of 3 independent experiments unless otherwise stated, with results represented as mean and standard error of the mean. A 2-way ANOVA with Dunnett’s multiple comparison test was used to analyze the linear quadratic survival curves in clonogenic assays. A 1-way ANOVA with Dunnett’s multiple comparison test was used to compare the tumor growth in CD1-nude mice. The Kaplan-Meier method was used to present the time to quadruple volume in tumors, and statistical significance (*P* < .05) was determined using the Mantel-Cox test.

## Results

### Romidepsin increases the histone acetylation level and causes cytotoxicity and radiosensitivity in bladder cancer cell lines

**Fig. 1:**
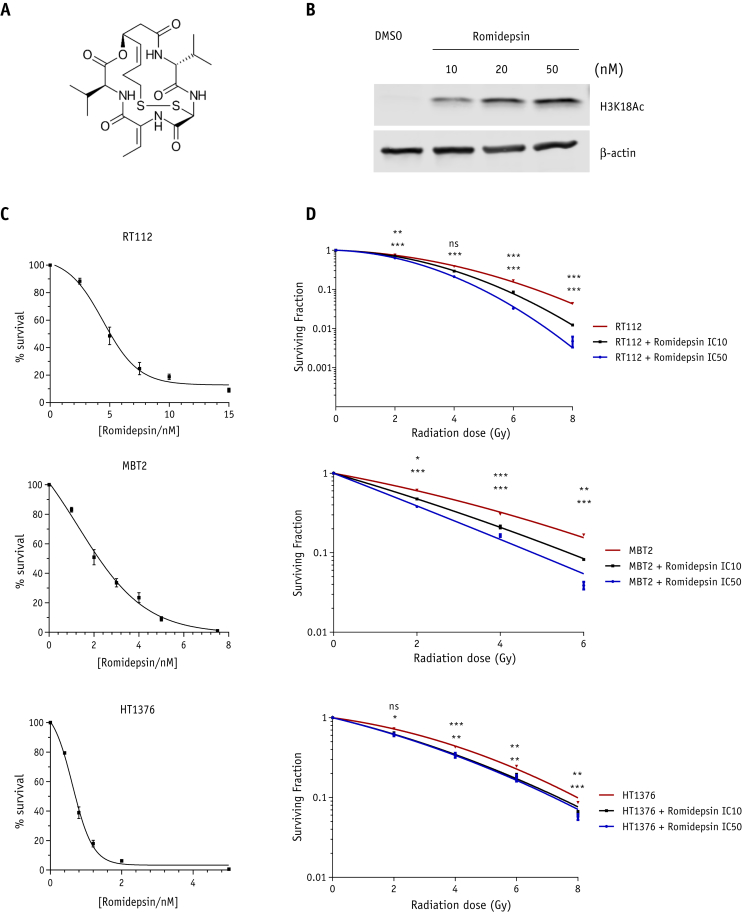
Romidepsin increases the level of histone 3 acetylation and causes cytotoxicity and radiosensitivity in bladder cancer cell lines. (A) Chemical structure of romidepsin; (B) dose-dependency analysis by Western blot of histone 3 acetylation level in RT112 cells. Equal loading is shown by β-actin; (C) survival curves of RT112, MBT2, and HT1376 cells after 24 hours of treatment with indicated concentrations of romidepsin to determine IC10 (RT112: 2.5 nM; MBT2: 0.6 nM; HT1376: 0.4 nM) and IC50 (RT112: 5 nM; MBT2: 2 nM; and HT1376: 0.6 nM). (D) Linear quadratic survival curves of RT112, MBT2, and HT1376 cells treated with ionizing radiation either in combination with romidepsin IC10 and IC50 or alone. Graphs represent 3 biological repeats over 3 technical replicates. All error bars represent ± standard error of the mean. *Abbreviation:* NS = not significant. *P < .05; **P < .01; ***P < .001.

Western blotting was performed to confirm that romidepsin resulted in histone acetylation in the RT112 bladder cancer cell line. Results showed that treatment with romidepsin acetylated histone H3 in a concentration-dependent manner (Fig. 1B).

The cytotoxicity of romidepsin was evaluated in RT112, MBT2, and HT1376 bladder cancer cell lines after treatment with increasing concentrations of romidepsin for 24 hours, and clonogenic survival was assessed. Romidepsin was effective in the nanomolar range in all bladder cancer cells (Fig. 1C). Survival curves were used to determine for each cell lines the IC10 (RT112: 2.5 nM; MBT2: 0.6 nM; HT1376: 0.4 nM) and the IC50 (RT112: 5nM; MBT2: 2nM; HT1376: 0.6nM). Subsequently, we investigated romidepsin as a radiosensitizer in RT112, MBT2, and HT1376 cells treated with ionizing radiation (IR), either in combination with romidepsin IC10 and IC50, or alone. Compared with control cells, romidepsin-treated cells were more radiosensitive, as shown by reduced clonogenic survival (Fig. 1D).

### Romidepsin increases growth delay in irradiated bladder cancer cell xenografts

**Fig. 2:**
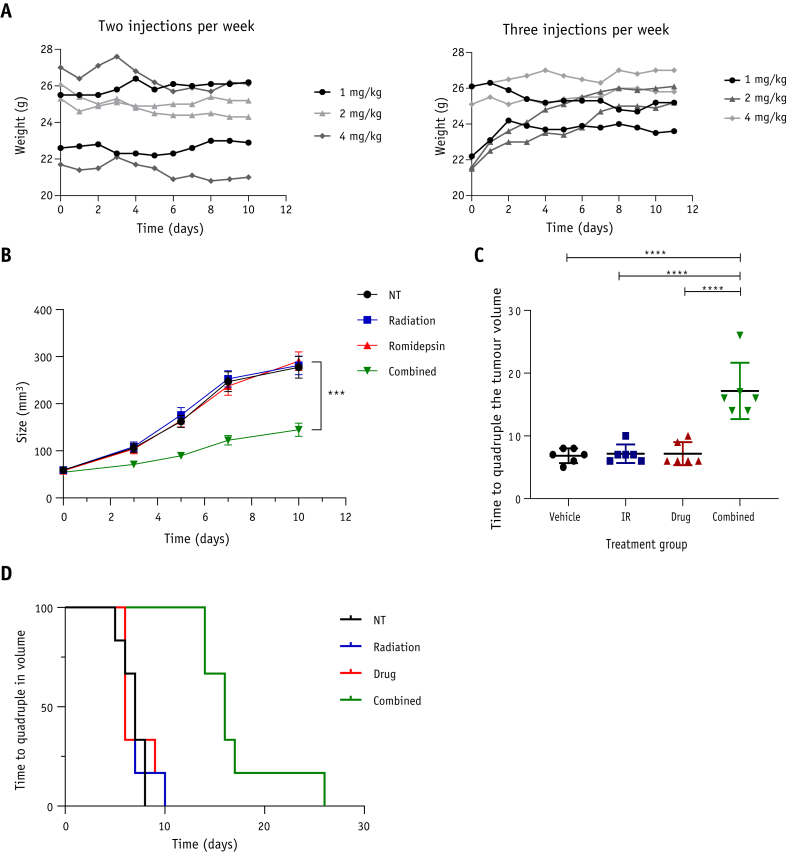
Romidepsin causes increased growth delay in irradiated bladder cancer cell xenografts and prolongs mouse survival. (A) Weight changes in 7-week-old CD1-nude mice after 2 (left panel) or 3 (right panel) intraperitoneal injections of 1 mg/kg (n = 2), 2 mg/kg (n = 2), or 4 mg/kg (n = 2). (B) RT112 tumor xenograft growth after treatment with vehicle, ionizing radiation (6 Gy, single dose), romidepsin (4 mg/kg, single injection), or romidepsin + ionizing radiation (n = 6 in each group). (C) Time to quadruple tumor volume after indicated treatments. D) Kaplan-Meier survival curve showing plots of time to quadruple tumor volume after indicated treatments. All error bars represent ± standard error of the mean. ***P < .001; ****P < .0001.

After establishing that romidepsin acts as a radiosensitizer in bladder cancer cell lines, we used an in vivo bladder cancer superficial xenograft model to confirm this effect. We first determined the maximum tolerated dose by injecting romidepsin intraperitoneally 2 or 3 times per week at a dose of 1 mg/kg, 2 mg/kg, or 4 mg/kg. We monitored body weight changes and found no significant body weight loss over the course of the experiment, demonstrating that the drug is well tolerated at these doses (Fig. 2A).

To test romidepsin as a radiosensitizer in vivo*,* RT112 cells were injected into the flank of CD1-nude mice (n = 6 per group), and mice were treated once xenografts reached 50 mm^3^. Mice treated with a combination of a single intraperitoneal injection of romidepsin at 4 mg/kg and radiation therapy (6 Gy, single fraction) showed significant tumor growth inhibition (*P* = .0002) compared with the control, IR-only, or drug-only groups (Fig. 2B). No significant difference was found between the control and IR- or drug-only groups. The time to quadruple tumor volume in the combined group was significantly different from the control, IR-only, or drug-only groups (17 ± 4.5 days vs 7 ± 1.5 days; *P* < .0001; Fig. 2C). The Kaplan-Meier curve for time to quadruple tumor volume was significantly prolonged in the romidepsin + IR group compared with all other groups (*P* = .007; Fig. 2D).

### Romidepsin does not increase acute intestinal toxicity after ionizing radiation

**Fig. 3:**
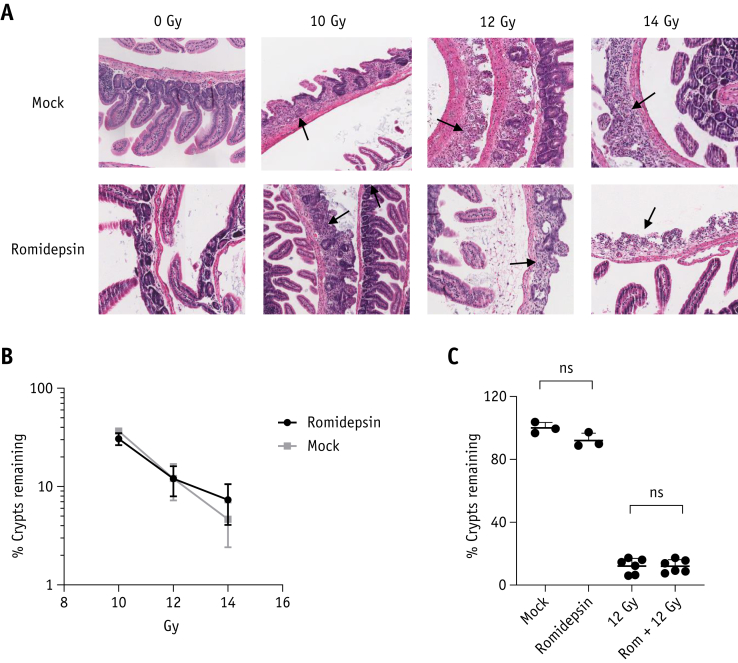
Romidepsin does not increase acute intestinal toxicity after ionizing radiation (IR). (A) Hematoxylin and eosin stained sections of small intestinal Swiss rolls demonstrating loss and regenerating crypts after 10, 12, and 14 Gy radiation ± romidepsin 4 mg/kg. (B) Small intestinal crypt assay survival for romidepsin and IR (n = 3 per group, except for 12 Gy IR: n = 6. Data were normalized to mean crypts per mm of 3 mock samples. (C) Effect on small intestinal crypt survival of vehicle (n = 3), romidepsin 4 mg/kg (n = 3), 12 Gy IR (n = 6), and romidepsin 4 mg/kg + 12 Gy IR (n = 6). All error bars represent ± standard error of the mean. *Abbreviation:* NS = not significant.

The effects of adding intraperitoneal romidepsin on acute radiation intestinal toxicity were tested in CD1-nude mice using a modified crypt assay, with irradiation to the lower abdomen only.^11^ No loss of small intestinal crypts was observed in mice treated with vehicle or drug alone (number of crypts per mm for mock: 18.7 ± 0.66 [n = 3]; romidepsin: 15.9 ± 1.39 [n = 3]; Fig. 3A). There was no significant difference in crypt loss between romidepsin + IR and IR alone at 10 Gy (*P* = .19), 12 Gy (*P* = .98), or 14 Gy (*P* = .34; Fig. 3: Fig. E1, available online at https://doi.org/10.1016/j.ijrobp.2020.01.015).

### Romidepsin does not increase long-term intestinal and bladder toxicity after ionizing radiation

Late radiation therapy complications can have a long-term impact on patients’ quality of life,^14^ whereas acute effects are usually time limited. We therefore assessed the potential late effects of romidepsin + IR compared with IR alone using a previously developed method.^11^ No significant difference was observed in body weight between IR and romidepsin + IR (Fig. E2A; available online at https://doi.org/10.1016/j.ijrobp.2020.01.015).

**Fig. 4:**
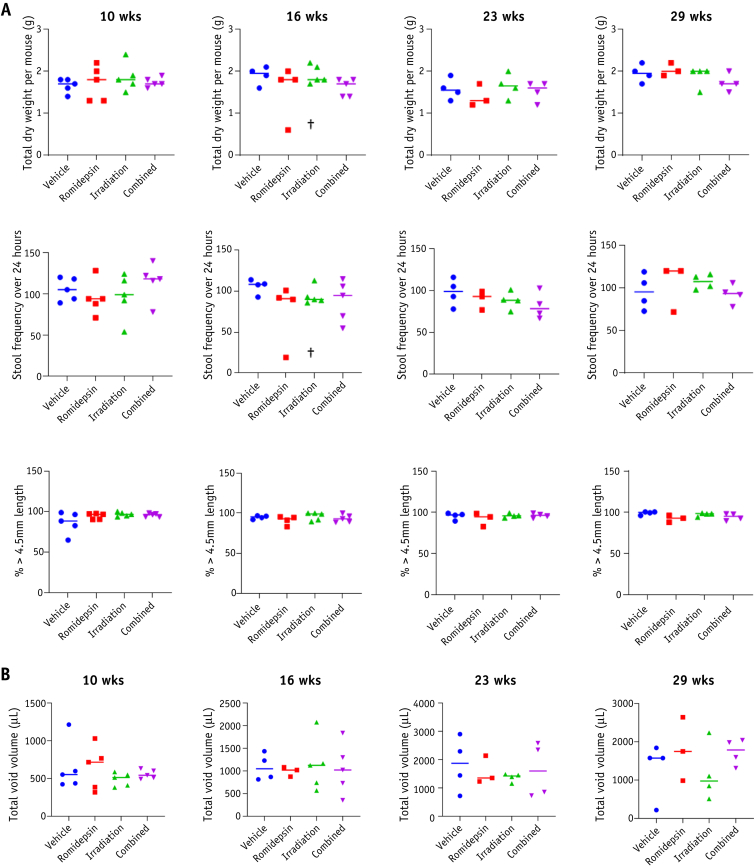
Romidepsin does not increase long-term intestinal and bladder toxicity after ionizing radiation. (A) Mice were isolated for 24 hours 10 weeks after treatment (n = 5 per group), 16 weeks after treatment (vehicle: n = 4, romidepsin: n = 4, radiation: n = 5, and combined: n = 5), and 23 and 29 weeks after treatment (vehicle: n = 4, romidepsin: n = 3, radiation: n = 4, and combined: n = 4). Feces were collected and weighed (upper panel), the total number of fecal pellets was counted (middle panel), and the percentage of fecal pellets ≥ 4.5 mm was assessed (lower). (B) Mice were isolated for 4 hours 10, 16, 23, and 29 weeks after treatment, and the total urinary voided volume was analyzed. ^†^Sick mice.

There was no significant difference in mean weight of dried feces or mean number of fecal pellets across the groups at 10, 16, 23, and 29 weeks and in all groups >85% of pellets were >4.5 mm in length (Fig. 4A; Table E1; available online at https://doi.org/10.1016/j.ijrobp.2020.01.015). Moreover, no significant difference was observed in fecal pellet length (Fig. S2B). A quantitative analysis of urinary voiding patterns showed no significant difference between the groups (Fig. 4B; Fig. E2C, available online at https://doi.org/10.1016/j.ijrobp.2020.01.015).

### Romidepsin impairs repair of DNA double-strand breaks after ionizing radiation

**Fig. 5:**
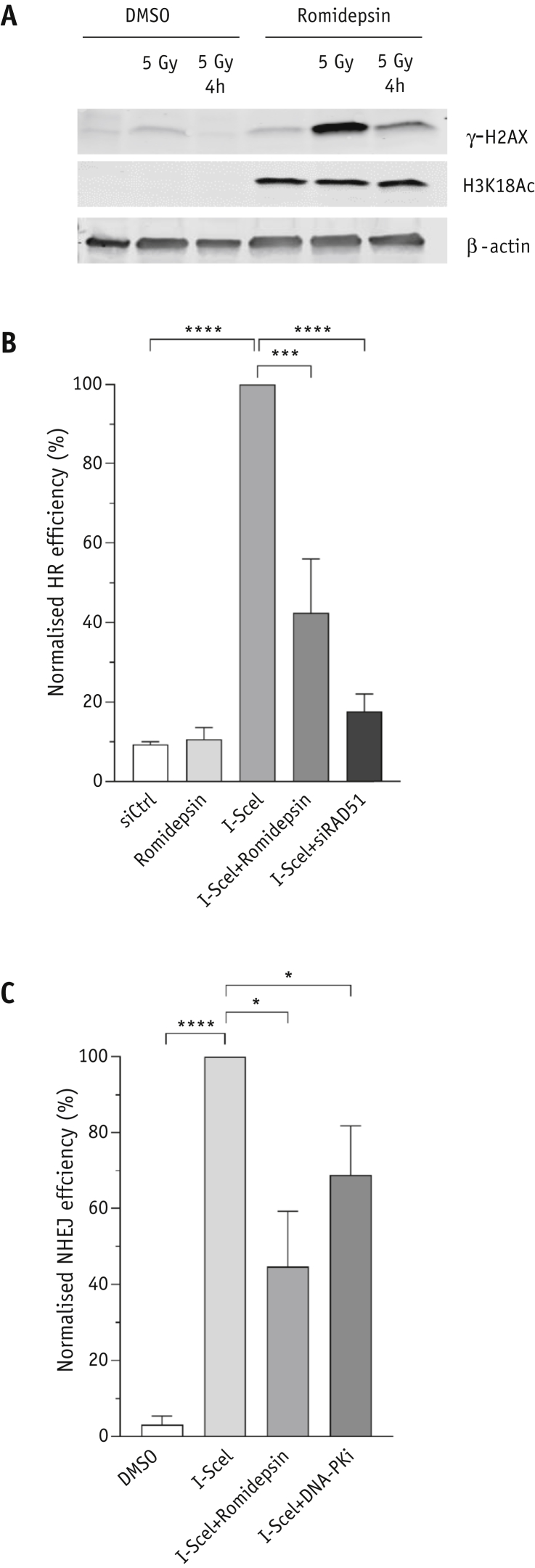
(A) Histone 3 acetylation and γ-H2AX level in RT112 cells treated with 5 Gy ionizing radiation either in combination with romidepsin or alone. (B) I-Sce1 assay for romidepsin (n = 3) and (C) nonhomologous end joining assay for romidepsin (n = 2). All error bars represent ± standard error of the mean. *P < .05; ***P < .001; ****P < .0001.

Ionizing radiation results in double-strand breaks (DSBs), which lead to the activation of the early DNA damage response.^15^ Impaired repair of DNA DSBs could be involved in the radiosensitivity in tumor cells after romidepsin treatment, as we already showed after panobinostat treatment.^11^^,^^16^ We assessed this response by measuring the level of phosphorylated H2AX (gamma-H2AX) in RT112 cells after 5 Gy IR ± romidepsin treatment. The results showed an increasing level of gamma-H2AX in control cells treated with dimethyl sulfoxide (DMSO) and in cells treated with romidepsin. However, the level was higher in romidepsin-treated cells than in control cells (Fig. 5A). Moreover, gamma-H2AX was still detected 4 hours after irradiation in romidepsin-treated cells but not in control cells, suggesting that DSBs are not repaired and accumulate with time in RT112 cells with romidepsin treatment.

The effects of romidepsin on the repair of IR-induced DSBs by HR and NHEJ were analyzed with direct repeat green fluorescent protein (DR-GFP) and EJ5-GFP reporter assays.^17^ We found that romidepsin treatment significantly decreased the percentage of GFP-positive cells in U2OS/DR-GFP cells after expression of I-SceI compared with cells without romidepsin treatment (Fig. 5B; *P* = .004). The same effect was observed in U2OS/EJ5-GFP cells (Fig. 5C; *P* < .05).

## Discussion

Muscle-invasive bladder cancer is therapeutically challenging, and there is an urgent need to find new effective chemotherapy agents for use with radiation therapy combinations that are less toxic to normal tissue and better tolerated by elderly patients.

Over the last decade, HDAC have emerged as important cancer therapeutic targets. High tumor expression levels are observed in high-grade urothelial bladder cancer.^7^ HDACi are epigenetic drugs that can modify histones and nonhistone proteins^18^ and are suitable for use as anticancer therapy^19^ because they have already shown promising effects in preclinical studies.^11^^,^^19^, ^20^, ^21^, ^22^ Several HDACi (ie, vorinostat, mocetinostat, belinostat) have been studied in clinical trials of urothelial bladder cancer.^23^ However, despite the clinical benefits of vorinostat, a pan-HDACi, it is reported to have serious adverse effects. Mocetinostat, class I and IV, is also compromised by severe toxicities.^24^ We previously published that IR + panobinostat delayed bladder tumor growth but did not increase acute and late normal tissue toxicity; however, panobinostat is a pan-HDACi.^11^ Thus, finding a more specific HDACi with a lesser extent of normal tissue toxicity in combination with radiation therapy might be better for patients. The HDACi romidepsin is a structurally unique, potent, bicyclic class I selective HDACi^25^ that could be an effective radiosensitizer with fewer systemic side effects than pan-HDAC inhibition.

In the present study, we have shown that romidepsin in combination with IR decreased the clonogenic survival of a panel of human bladder tumor cell lines and achieved tumor growth delay over radiation therapy alone. Most significantly, treatment with romidepsin + IR did not increase normal tissue toxicity caused by radiation to the surrounding normal bowel incorporated in the radiation field, either acutely at 3.75 days after radiation using a small intestinal crypt assay or later at 29 weeks based on functional bowel and bladder assays. Of note, romidepsin radiosensitizes bladder cancer in nanomolar concentrations, whereas mocetinostat, TMP195,^11^ and SAHA^26^ do so at micromolar levels. Romidepsin is already in clinical use for cutaneous T-cell lymphoma, but a phase 1 clinical trial of romidepsin as a radiosensitizer could be considered in muscle-invasive bladder cancer.

In addition, this study revealed that romidepsin treatment increased the level of gamma-H2AX protein immediately after IR, and this was still detectable 4 hours after IR compared with the control cells, suggesting that DSBs were not repaired and accumulated over time after romidepsin treatment. The enhanced tumor radiation response after treatment with HDACi, could be due to the modulation of DNA damage signaling and repair by HDACi.^27^, ^28^, ^29^, ^30^ Mammalian cells are widely known to rely mainly on the HR and NHEJ mechanisms to repair DNA DSBs.^31^, ^32^, ^33^

## Conclusions

We showed that romidepsin treatment impaired both HR and NHEJ DNA repair pathways, supporting evidence in the literature that HDACi may radiosensitize by partly suppressing DNA repair pathways.^34^, ^35^, ^36^ The demonstration of impaired repair by both NHEJ and HR caused by romidepsin raises the possibility that romidepsin may regulate proteins or processes common to both pathways. Further investigation of the molecular basis for these effects will shed light on the role of romidepsin in DNA repair pathways.

## Supplementary Data

Fig E1

Fig E2

Supplementary Material

Table E1
