# Supplementary figures and images for: The Histone Deacetylase Inhibitor Romidepsin Spares Normal Tissues While Acting as an Effective Radiosensitizer in Bladder Tumors in Vivo
Source: Int J Radiat Oncol Biol Phys. 2020 May 1;107(1):212–21. doi: 10.1016/j.ijrobp.2020.01.015 (PMC7181176; doi:10.1016/j.ijrobp.2020.01.015)

Supplementary figure 1

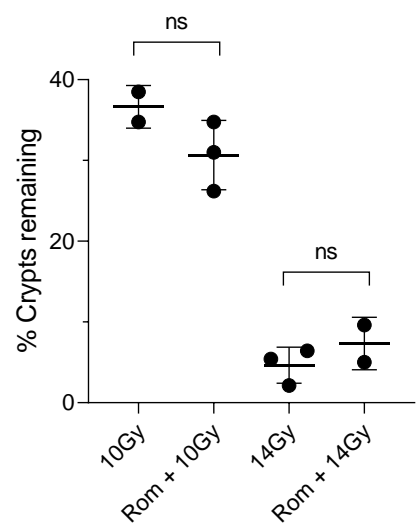

Supplement: Fig E1 [file mmc1.pdf]

Supplementary figure 2

A

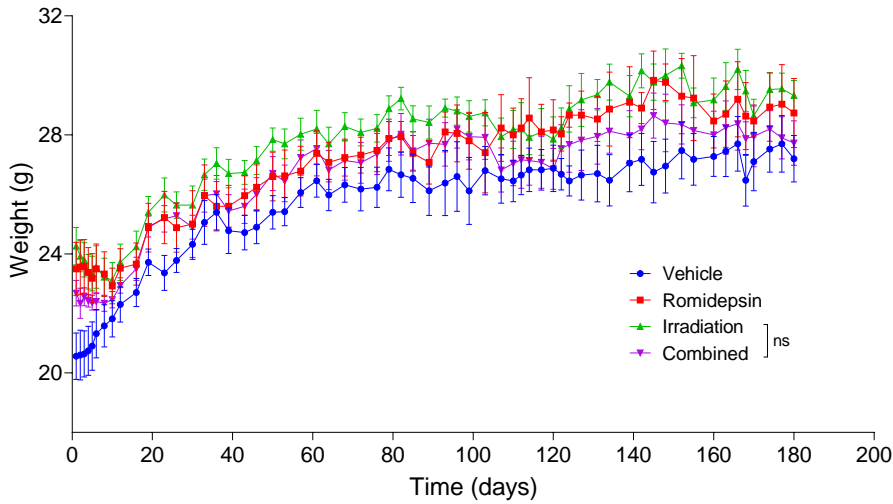

B

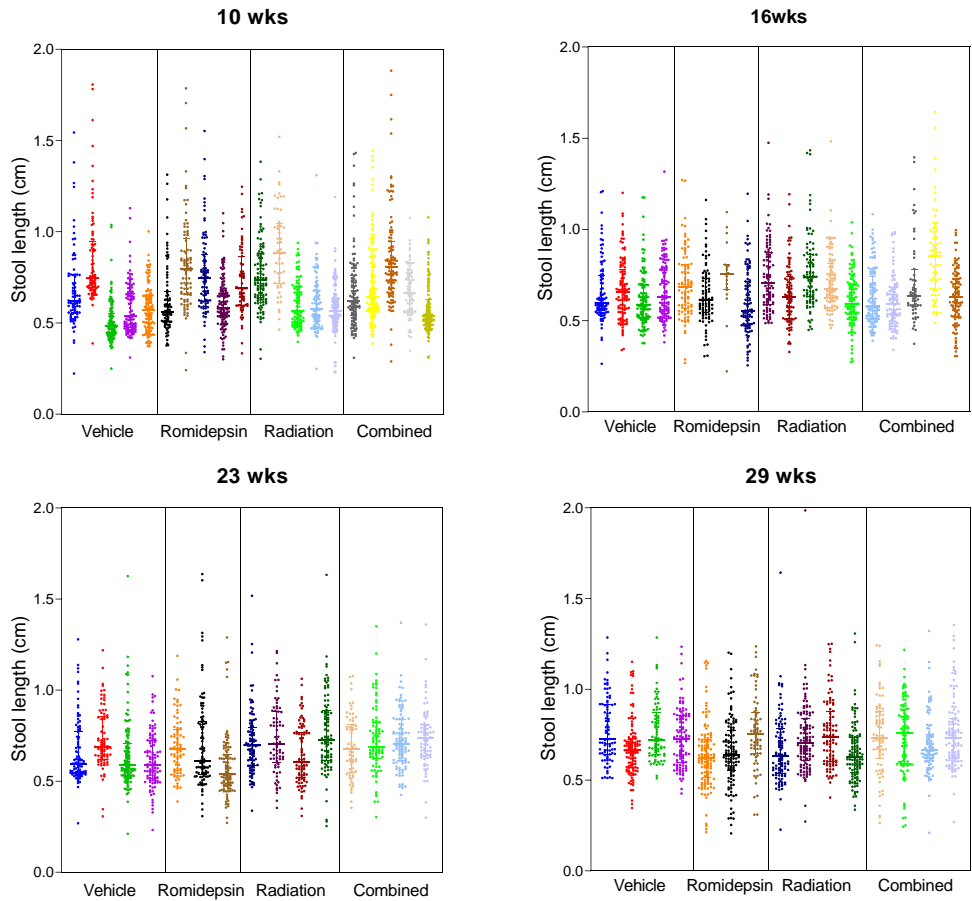

C

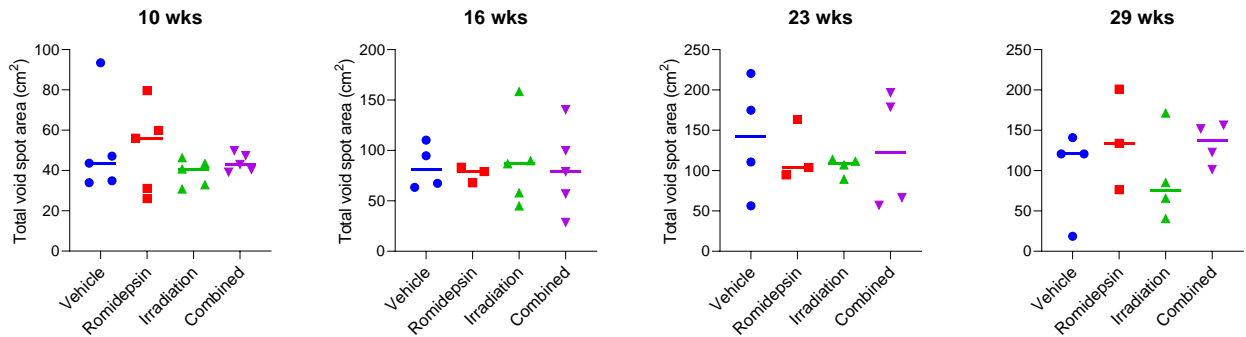

Supplement: Fig E2 [file mmc2.pdf]
