# Supplementary material for: The Histone Deacetylase Inhibitor Romidepsin Spares Normal Tissues While Acting as an Effective Radiosensitizer in Bladder Tumors in Vivo
Source: Int J Radiat Oncol Biol Phys. 2020 May 1;107(1):212–21. doi: 10.1016/j.ijrobp.2020.01.015 (PMC7181176; doi:10.1016/j.ijrobp.2020.01.015)
Supplement: Supplementary Material [file mmc3.docx]

**Supplementary Material**

**Supplementary Methods**

**Colony formation assay**

Colonies were fixed and stained with 0.5% crystal violet in dH_2_O and 20% methanol for 5-10 minutes and quantified using a GelCount colony counter (Oxford Optronix). The surviving fraction was determined by normalising the number of colonies in each condition to the unirradiated control. For the drug-only clonogenic assay, cells were normalised to DMSO control. All experiments were conducted in triplicate across 3 technical replicates.

**Acute toxicity**

Six to seven-week-old female CD1-nude mice were treated with vehicle (5% DMSO in dH_2_O, intraperitoneally) or romidepsin (4 mg/kg, intraperitoneally). Approximately 6 hours later, mice were treated supine with 10, 12, or 14 Gy of x-rays (220 kV_p_, 13.0 mA copper filtered beam with a measured HVL of 0.84 mmCu) to the lower abdomen, including the lower small intestine using a Small Animal Radiation Research Platform (SARRP) irradiator (Xstrahl Ltd, Camberley, UK), using a 356-degree arc treatment with a 13.7-mm circular collimator (at isocentre) at a dose rate of approximately 2 Gy per minute. Dosimetry commissioning for the treatment planning software was performed using Gafchromic EBT3 Film (Ashland Advanced Materials, Bridgewater NJ) (1,2), which was calibrated following the recommendations of the report of AAPM Task Group 61 (3) and traceable to the UK standard at the National Physical Laboratory. Throughout this procedure, mice were anaesthetised (1-3% isoflurane in ca. 30% O_2_-enriched air) using a custom-made 3D printed cradle that featured skull immobilisation, gas anaesthetic delivery, respiration monitoring and rectal temperature maintenance at 35-37^o^C (4). Mice were sacrificed at 3.75 days and tissue collected. Swiss rolls (5) were made from large (one length) and small intestines (three consecutive lengths). The samples were formalin-fixed overnight in 10% neutral buffered formalin and stored in 70% ethanol prior to paraffin-embedding. Four micrometre sections were cut and stained for haematoxylin and eosin (H&E) and analysed in a crypt assay as previously described (5).

***Long-term toxicity***

Six to seven-week-old CD1-nude mice were treated with vehicle (5% DMSO in dH_2_O, intraperitoneally) or Romidepsin (4 mg/kg, intraperitoneally). Approximately 6 hours later, mice were treated using a SARRP with 5 Gy for 5 consecutive days, using a 356-degree arc treatment and 8.5-mm collimator, with the isocentre positioned at the posterior caudal bladder wall, to avoid the small intestine. Throughout this procedure mice were anaesthetised (1-3% isoflurane in ca. 30% O2-enriched air), and treated vertically, head down in a custom-made 3D printed cradle that featured skull immobilisation, anaesthetic delivery, respiration monitoring and rectal temperature maintenance at 35-37^o^C (4). Mice have been checked for behaviours and weighed daily for the first 5 days, then 3 times a week the following week and then twice a week during the rest of the whole experiment period. Mice were singly housed for 24 hours in week 10, 16, 23 and 29 post-irradiation, for stool collection and analysis (weighed, counted, and measured) and 4 hours for urinary frequency assessment. The spontaneous urinary voiding by mice was assessed as previously described (6).

**Crypt assay**

H&E-stained Swiss roll slides were scanned using an Aperio CS2 digital pathology scanner (Leica). As only part of the abdomen had been irradiated, the most affected areas in each Swiss roll were selected by two independent observers. Irradiated mouse samples were excluded if there was less than 3 mm of intestinal damage. Regenerating crypts (presence of >10 cells arranged in a distinct shape with no sign of apoptosis) were blind counted by two independent observers. The control number of crypts per length of small intestine, was determined from the mean of three mock-treated mice. The percentage of surviving crypts in each group was calculated as:

Number of regenerating crypts per mm x 100

Number of control crypts per mm

**References**

1. E. Tryggestad, M. Armour, I. Iordachita, F. Verhaegen and J.W. Wong, A comprehensive system for dosimetric commissioning and Monte Carlo validation for the small animal radiation research platform, *Phys Med Biol* **54**, 2009, 5341–5357

2. M.A. Hill, J.M. Thompson, A. Kavanagh, et al., The development of technology for effective respiratory-gated irradiation using an image-guided small animal irradiator. *Radiat Res* **188**, 2017, 247-263.

3. C.M. Ma, C.W. Coffey, L.A. DeWerd, et al., AAPM protocol for 40–300 kV x-ray beam dosimetry in radiotherapy and radiobiology, *Med Phys* **28**, 2001, 868–893.

4. V. Kerseman, S. Gilchrist, S. Wallington, et al., A carbon-fiber sheet resistor for MR-, CT-, SPECT-, and PET-compatible temperature maintenance in small animals. *Tomography* **5**, 2019, 274-281.

5. B. Groselj, J-L. Ruan, H. Scott, et al., Radiosensitisation in vivo by histone deacetylase inhibition with no increase in early normal tissue radiation toxicity. *Mol Cancer Ther* **17**, 2018, 381-392.

6. W. Yu, C. Ackert-Bicknell, J.D. Larigakis, et al., Spontaneous voiding by mice reveals strain-specific lower urinary tract function to be a quantitative genetic trait, *Am J Physiol Renal Physiol* **306**, 2014, 1296–1307.

**Supplementary Figure 1.** Effect on small intestinal crypts of 10 Gy IR (n = 2), romidepsin 4 mg/kg + 10 Gy IR (n = 3), 14 Gy IR (n = 3) and romidepsin 4 mg/kg + 14 Gy IR (n = 2). Data were normalised to mean crypts of three mock samples. All error bars represent +/- SEM. NS, not significant.

**Supplementary Figure 2.** A, Mean body weight over the duration of the experiment in vehicle, romidepsin, IR and romidepsin + IR groups .B, Mice were isolated for 24 hours 10 weeks after treatment (n = 5 per group), 16 weeks after treatment (vehicle n = 4, romidepsin n = 4, radiation n = 5 and combined n = 5) and 23 weeks after treatment (vehicle n = 4, romidepsin n = 3, radiation n = 4 and combined n = 4) and their faeces collected for length measurement. C, Mice were isolated for 4 hours 10, 16 and 23 weeks after treatment, and the total urinary void spot area was assessed.

**Supplementary Table 1.** Mice were isolated for 24 hours 10 weeks after treatment (n = 5 per group), 16 weeks after treatment (vehicle n = 4, romidepsin n = 4, radiation n = 5 and combined n = 5), 23 and 29 weeks after treatment (vehicle n = 4, romidepsin n = 3, radiation n = 4 and combined n = 4). Their faeces were collected the total number of faecal pellets per mouse was counted.
