# Supplementary material for: The Histone Deacetylase Inhibitor Romidepsin Spares Normal Tissues While Acting as an Effective Radiosensitizer in Bladder Tumors in Vivo
Source: Int J Radiat Oncol Biol Phys. 2020 May 1;107(1):212–21. doi: 10.1016/j.ijrobp.2020.01.015 (PMC7181176; doi:10.1016/j.ijrobp.2020.01.015)
Supplement: Table E1 [file mmc4.docx]

**Supplementary Table 1. Total number of faecal pellets per mouse.** Mice were isolated for 24 hours 10 weeks after treatment (n=5 per group), 16 weeks after treatment (vehicle n=4, romidepsin n=4, radiation n=5 and combined n=5), 23 and 29 weeks after treatment (vehicle n=4, romidepsin n=3, radiation n=4 and combined n=4). Their faeces were collected and the total number of faecal pellets per mouse was counted.

|  | **10 weeks** | | | | **16 weeks** | | | |
| --- | --- | --- | --- | --- | --- | --- | --- | --- |
|  | **Vehicle** | **Romidepsin** | **Irradiation** | **Combined** | **Vehicle** | **Romidepsin** | **Irradiation** | **Combined** |
|  | 89 | 88 | 116 | 140 | 93 | 90 | 93 | 115 |
|  | 94 | 94 | 54 | 118 | 109 | 92 | 86 | 95 |
|  | 120 | 94 | 92 | 116 | 114 | 19 | 90 | 55 |
|  | 105 | 128 | 99 | 78 | 108 | 101 | 89 | 70 |
|  | 118 | 71 | 124 | 122 |  |  | 113 | 106 |
| Average | 105.2 | 95.0 | 97.0 | 114.8 | 106.0 | 75.5 | 94.2 | 88.2 |
| STD | 13.9 | 20.7 | 27.2 | 22.7 | 9.1 | 38.0 | 10.8 | 25.1 |
|  |  |  |  |  |  |  |  |  |
|  | **23 weeks** | | | | **29 weeks** | | | |
|  | **Vehicle** | **Romidepsin** | **Irradiation** | **Combined** | **Vehicle** | **Romidepsin** | **Irradiation** | **Combined** |
|  | 93 | 93 | 88 | 67 | 73 | 120 | 113 | 106 |
|  | 116 | 77 | 75 | 84 | 85 | 72 | 116 | 78 |
|  | 105 | 99 | 101 | 73 | 119 | 120 | 98 | 95 |
|  | 78 |  | 89 | 103 | 106 |  | 102 | 92 |
| Average | 98.0 | 89.7 | 88.3 | 81.8 | 95.8 | 104.0 | 107.3 | 92.8 |
| STD | 16.3 | 11.4 | 10.6 | 15.8 | 20.6 | 27.7 | 8.6 | 11.5 |
